# Supplementary material for: Diversity of Mycorrhizal Fungi in Temperate Orchid Species: Comparison of Culture-Dependent and Culture-Independent Methods
Source: J Fungi (Basel). 2024 Jan 23;10(2):92. doi: 10.3390/jof10020092 (PMC10890429; doi:10.3390/jof10020092)
Supplement: Supplementary file 1 [file jof-10-00092-s001.zip › jof-2819066-supplementary.pdf]

Supplementary information for:

Diversity of Mycorrhizal Fungi in Temperate Orchid Species: Comparison of Culture-Dependent and Culture-Independent Methods

Sophie Mennicken [1], Caio César Pires de Paula [1,2], Héléne Vogt-Schilb [1,3] and Jana Jersáková [1,*]

Supplementary tables and figures:

Table S1. Rhizoctonian operational taxonomic units (OTUs) obtained in the study.

Table S2. Remaining fungal OTUs belonging to non-rhizoctonia families obtained by NGS method.

Table S3. Most abundant fungal OTUs and their cultivability among the orchid species.

Table S4. ANOVA multiple comparisons of the number of OTUs (fungal richness) among orchid plants.

Table S5. Distance-based Redundance analysis results on the effect of locality and orchid host identity on the fungal communities in plants of 11 orchid species that occurred at least in two sites.

Table S6. ANOVA multiple comparisons of the mean Shannon, intersite and interspecies dissimilarity indices of rhizoctonian fungal communities.

Table S7. Details on the number of fungal OTUs, plants, roots, and culture plates in each studied orchid species.

Figure S1. Rhizoctonian OTUs at individual plant level in the 19 studied orchid species.

Supplementary tables:

Table S1. Rhizoctonian operational taxonomic units (OTUs) obtained in the study. Details on families and GenBank accession numbers of the 54 fungal OTUs[a] and their corresponding 30 fungal strains obtained by peloton cultivation followed by Sanger sequencing. The strains with the symbol (*) were not cultured in 2018, but in previous unpublished projects. See phylogenetic trees (Figure 4).

OTUs code	Family	Number of sequence reads	Fungal OTUs GenBank Accession no.	Corresponding fungal strains	Isolates' code	Fungal strains GenBank Accession no.
C1	Ceratobasidiaceae	67098	OP739278	CER16	CER_16_P36_PB	OR990580
C2	Ceratobasidiaceae	39461	OP739279	CER6	CER_6_J143_PB	OR990571
C3	Ceratobasidiaceae	24439	OP739280	CER9	CER_9_Z12_PB	OR990573
C4	Ceratobasidiaceae	19083	OP739281	CER10	CER_10_Z125_AP	OR990574
C5	Ceratobasidiaceae	14023	OP739282	CER19	CER_19_C188_OPP	MZ503000
C6	Ceratobasidiaceae	6134	OP739283	CER1	CER_1_C209_NU	MZ502998
C7	Ceratobasidiaceae	5188	OP739284	CER12	CER_12_C208_NU	OR990576
C8	Ceratobasidiaceae	3432	OP739285	CER17	CER_17_C126_AM	MZ502999
C9	Ceratobasidiaceae	2057	OP739286	CER11	CER_11_P4_DV	OR990575
C10	Ceratobasidiaceae	1948	OP739287	CER13	CER_13_P127_AP	OR990577
C11	Ceratobasidiaceae	1605	OP739288	Uncultured	-	-
C12	Ceratobasidiaceae	1422	OP739289	CER15	CER_15_C218_AP	OR990579
C13	Ceratobasidiaceae	1032	OP739290	Uncultured	-	-
C14	Ceratobasidiaceae	538	OP739291	CER14	CER_14_P151_OPP	OR990578
C15	Ceratobasidiaceae	525	OP739292	CER18	CER_18_C119_AM	OR990581
C16	Ceratobasidiaceae	474	OP739293	CER20	CER_20_C197_OPP	OR990582
C17	Ceratobasidiaceae	412	OR897826	Uncultured	-	-
C18	Ceratobasidiaceae	230	OP739294	CER7*	OTU9_CER_7_DM34 and OTU9_J	OR990572
C19	Ceratobasidiaceae	223	OR897825	CER2*	CER_2N_AP12A-P15	MK951653
S1	Serendipitaceae	48752	OP739299	SEB4	SEB_4_J334_NO	MZ503002
S2	Serendipitaceae	19483	OP739300	Uncultured	-	-
S3	Serendipitaceae	12314	OP739302	SEB2*	SEB_2_ZNO23	OR990583
S4	Serendipitaceae	5065	OP739303	Uncultured	-	-
S5	Serendipitaceae	829	OP739304	Uncultured	-	-
S6	Serendipitaceae	311	OP739305	Uncultured	-	-
S7	Serendipitaceae	7	OP739306	Uncultured	-	-
S8	Serendipitaceae	3	OP739307	Uncultured	-	-
S9	Serendipitaceae	3	OP739308	SEB3	SEB_3_J329_NO	MZ503001
-	Serendipitaceae	-	-	SEB1*	SEB_1_DAMA9	MK951654
T1	Tulasnellaceae	305557	OP739309	Uncultured	-	-
T2	Tulasnellaceae	131432	OP739310	TUL4	TUL_4_C182_OMA	MZ503004

T3	Tulasnellaceae	128782	OP739311	TUL1	TUL_1_Z210_AP	MZ503003
T4	Tulasnellaceae	121921	OP739312	TUL7	TUL_7_J146_PB	MZ503005
T5	Tulasnellaceae	104277	OP739313	Uncultured	-	-
T6	Tulasnellaceae	86876	OP739314	Uncultured	-	-
T7	Tulasnellaceae	67297	OP739315	TUL11	TUL_11_Z165_NO	OR990585
T8	Tulasnellaceae	52418	OP739316	TUL14	TUL_14_Z213_GM	OR990587
T9	Tulasnellaceae	40696	OP739317	TUL8	TUL_8_P52_DS	MZ503006
T10	Tulasnellaceae	37364	OP739318	Uncultured	-	-
T11	Tulasnellaceae	37225	OP739319	TUL12	TUL_12_Z155_OPA	OR990586
T12	Tulasnellaceae	29300	OP739320	TUL9	TUL_9_J55_GC	OR990584
T13	Tulasnellaceae	27577	OP739321	TUL16	TUL_16_Z180_OPH	OR990589
T14	Tulasnellaceae	20569	OP739322	TUL18	TUL_18_P164_NU	OR990591
T15	Tulasnellaceae	18279	OP739323	Uncultured	-	-
T16	Tulasnellaceae	12022	OP739324	TUL10*	OTU24_VJ7-2a-konOFTul	KC243955
T17	Tulasnellaceae	10686	OP739325	Uncultured	-	-
T18	Tulasnellaceae	9859	OR897823	TUL20	TUL_20_Z129_AP	OR990593
T19	Tulasnellaceae	9197	OP739326	Uncultured	-	-
T20	Tulasnellaceae	8616	OP739327	TUL19	TUL_19_P214_NO	OR990592
T21	Tulasnellaceae	1814	OR897822	Uncultured	-	-
T22	Tulasnellaceae	1732	OP739328	TUL17	TUL_17_Z187_AP	OR990590
T23	Tulasnellaceae	284	OR897821	TUL15	TUL_15_Z205_NU	OR990588
T24	Tulasnellaceae	164	OP739329	Uncultured	-	-
T25	Tulasnellaceae	6	OR897820	Uncultured	-	-
T26	Tulasnellaceae	2	OP739330	Uncultured	-	-

[a] Fungi were grouped into OTUs defined by 97% internal transcribed spacer (ITS) sequence similarity, using next-generation sequencing.

Table S2. Remaining fungal OTUs belonging to non-rhizoctonia families obtained by NGS method. Fungal OTUs of six families with potential mycorrhizal abilities (see Dearnaley et al., 2012), their orchid hosts, site of occurrence, number of sequence reads and corresponding relative frequencies, and GenBank accession numbers. The orchid species include *Anacamptis morio* (AM); *Dactylorhiza sambucina* (DS), *D. viridis* (DV); *Gymnadenia conopsea* diploid (GCd); *Neotinea ustulata* (NU); *Neottia ovata* (NO); *Orchis anthropophora* (OA), *O. mascula* (OMA), *O. militaris* (OMI); *Ophrys sphegodes subsp. passionis* (OPP) and *Platanthera spp.* (PL).

OTUs code	Family	Site	No. of orchid hosts	Orchid species code	No. of reads	Relative frequencies [0-1]	Fungal strains GenBank Accession no.
M1	Mycenaceae	CZ1	1	GCd	8	0.08	OR897824
O1	Omphalotaceae	CZ1	1	GCd	56	0.28	OP739295
P1	Psathyrellaceae	FR1	1	OMI	8	0.13	OP739296
R1	Russulaceae	CZ1	1	GCd	578	0.47	OP739297
R2	Russulaceae	CZ1	1	GCd	4	0.11	OP739298
SB1	Sebacinaceae	FR1	4	GCd; DS; NO; OPP	13946	0.11 - 1	OP739301
TH1	Thelephoraceae	FR1	7	GCd; DV; AM; PL; OMA; OPP; NU	67	0.05 - 0.24	OP739331
		FR2	2	AM; NU		0.06 - 0.17	
TH2	Thelephoraceae	FR2	1	OA	42	0.22	OP739332
TH3	Thelephoraceae	FR2	3	OA; AM; OMA	9	0.08 - 0.13	OP739333

Table S3. Most abundant fungal OTUs and their cultivability among the orchid species. The 24 most abundant OTUs with at least 20 % of relative frequencies of sequence reads in their orchid hosts across the four sites (FR1, FR2 in France and CZ1, CZ2 in the Czech Republic). The symbol (°) indicates the 4 abundant OTUs that failed in cultivation across all studied orchids.

OTUs (NGS)	Orchid hosts	Sites	Abundance	Cultivability	Corresponding strains
C1	*Platanthera spp.* (PL)	FR1	0.78	Cultured	CER16
C1	*Platanthera spp.* (PL)	FR2	0.80	Cultured	CER16
C1	*Platanthera bifolia* (PB)	CZ2	0.20	Cultured	CER16
C2	*Platanthera bifolia* (PB)	CZ1	0.59	Cultured	CER6
C3	*Platanthera bifolia* (PB)	CZ2	0.53	Cultured	CER9
C4	*Neotinea ustulata* (NU)	CZ2	0.45	Cultured	CER10
C5	*Neotinea ustulata* (NU)	FR2	0.54	Cultured	CER19
C7	*Dactylorhiza sambucina* (DS)	FR1	0.49	Not Cultured	CER12
C8	*Anacamptis morio* (AM)	FR2	0.24	Cultured	CER17
C9	*Dactylorhiza viridis* (DV)	FR1	0.50	Cultured	CER11

S1	*Neottia ovata* (NO)	CZ1	0.64	Cultured	SEB4
S1	*Neottia ovata* (NO)	CZ2	0.25	Not Cultured	SEB4
T1°	*Orchis militaris* (OMI)	FR2	0.94	Not Cultured	none
T1°	*Orchis purpurea* (OPU)	FR2	0.97	Not Cultured	none
T1°	*Orchis purpurea* (OPU)	FR1	0.73	Not Cultured	none
T1°	*Orchis militaris* (OMI)	FR1	0.75	Not Cultured	none
T2	*Orchis mascula* (OMA)	FR1	0.84	Cultured	TUL4
T2	*Orchis mascula* (OMA)	FR2	0.94	Cultured	TUL4
T2	*Orchis simia* (OSI)	FR2	0.22	Not Cultured	TUL4
T3	*Anacamptis pyramidalis* (AP)	FR1	0.46	Cultured	TUL1
T3	*Anacamptis pyramidalis* (AP)	FR2	0.74	Cultured	TUL1
T3	*Ophrys sphegodes subsp. passionis* (OPP)	FR2	0.49	Cultured	TUL1
T3	*Anacamptis pyramidalis* (AP)	CZ2	0.61	Cultured	TUL1
T4	*Dactylorhiza viridis* (DV)	FR1	0.30	Cultured	TUL7
T4	*Anacamptis morio* (AM)	CZ1	0.52	Cultured	TUL7
T4	*Gymnadenia conopsea*, diploid (GCd)	CZ1	0.48	Cultured	TUL7
T4	*Platanthera bifolia* (PB)	CZ1	0.21	Cultured	TUL7
T4	*Neotinea ustulata* (NU)	CZ2	0.23	Cultured	TUL7
T4	*Dactylorhiza sambucina* (DS)	CZ1	0.27	Not Cultured	TUL7
T5°	*Orchis anthropophora* (OA)	FR2	0.62	Not Cultured	none
T5°	*Orchis simia* (OSI)	FR2	0.42	Not Cultured	none
T6°	*Orchis militaris* (OMI)	CZ2	0.60	Not Cultured	none
T7	*Gymnadenia conopsea*, diploid (GCd)	CZ2	0.69	Cultured	TUL11
T7	*Neottia ovata* (NO)	CZ2	0.45	Cultured	TUL11
T7	*Gymnadenia densiflora* (GD)	CZ2	1	Not Cultured	TUL11
T8	*Gymnadenia conopsea*, tetraploid (GCt)	CZ2	0.22	Cultured	TUL14
T8	*Gymnadenia conopsea*, diploid (GCd)	FR1	0.59	Not Cultured	TUL14
T9	*Anacamptis morio* (AM)	FR1	0.49	Cultured	TUL8
T9	*Dactylorhiza sambucina* (DS)	FR1	0.34	Cultured	TUL8
T9	*Anacamptis morio* (AM)	FR2	0.21	Cultured	TUL8
T11	*Gymnadenia conopsea*, tetraploid (GCt)	CZ2	0.48	Cultured	TUL12
T11	*Ophrys apifera* (OPA)	CZ2	0.95	Cultured	TUL12
T12	*Anacamptis morio* (AM)	CZ1	0.24	Cultured	TUL9
T12	*Dactylorhiza sambucina* (DS)	CZ1	0.28	Cultured	TUL9
T12	*Gymnadenia conopsea*, diploid (GCd)	CZ1	0.29	Cultured	TUL9
T13	*Ophrys holubyana* (OPH)	CZ2	0.74	Cultured	TUL16
T14	*Neotinea ustulata* (NU)	FR1	0.51	Cultured	TUL18
T14	*Ophrys sphegodes subsp. passionis* (OPP)	FR1	0.44	Cultured	TUL18
T14	*Ophrys sphegodes subsp. passionis* (OPP)	FR2	0.23	Not Cultured	TUL18
T15°	*Ophrys sphegodes subsp. passionis* (OPP)	FR1	0.42	Not Cultured	none
T16	*Dactylorhiza sambucina* (DS)	CZ1	0.38	Not Cultured	TUL10
T20	*Orchis simia* (OSI)	FR2	0.21	Not Cultured	TUL19

Table S4. ANOVA multiple comparisons of the number of OTUs (fungal richness) among orchid plants. A. Anova tests comparing the fungal richness between orchid species, **B.** Tukey's Post-hoc tests comparing the fungal richness between orchid species across all sites.

A. Anova test on sites comparison

Number of OTUs	Df	Sum Sq	Mean Sq	F value	P value	Sign.
Species	18	70.1	3.89	3.15	**2.49e-05**	***
Residuals	289	357.3	1.24			

B. Tukey multiple comparisons between orchid species

Species	Estimate	Std.Error	t value	P value	Sign.
AP - AM	-0.54	0.29	-1.87	0.93	
DS - AM	-0.89	0.37	-2.44	0.58	
DV - AM	-0.76	0.54	-1.41	1.00	
GCd - AM	-0.71	0.30	-2.38	0.63	
GD - AM	-1.96	0.60	-3.30	0.10	.
GCt - AM	-0.96	0.43	-2.25	0.72	
NO - AM	-0.49	0.33	-1.47	0.99	
NU - AM	-0.10	0.32	-0.31	1.00	
OA - AM	-1.19	0.43	-2.77	0.33	
OMA - AM	-1.59	0.35	-4.53	**<0.01**	**
OMI - AM	-1.20	0.29	-4.18	**<0.01**	**
OPA - AM	-1.46	0.50	-2.92	0.25	
OPH - AM	-0.85	0.43	-2.00	0.88	
OPP - AM	-0.90	0.34	-2.63	0.44	
OPU - AM	-1.30	0.34	-3.83	**0.02**	*
OSI - AM	-0.71	0.45	-1.59	0.98	
PB - AM	-1.10	0.36	-3.06	0.18	
PL - AM	-1.46	0.35	-4.17	**<0.01**	**
DS - AP	-0.35	0.35	-1.00	1.00	
DV - AP	-0.22	0.53	-0.42	1.00	
GCd - AP	-0.17	0.29	-0.61	1.00	
GD - AP	-1.42	0.59	-2.42	0.60	
GCt - AP	-0.42	0.42	-1.02	1.00	
NO - AP	0.05	0.32	0.15	1.00	
NU - AP	0.44	0.31	1.44	0.99	
OA - AP	-0.65	0.42	-1.55	0.99	
OMA - AP	-1.05	0.34	-3.10	0.16	
OMI - AP	-0.67	0.27	-2.44	0.58	
OPA - AP	-0.92	0.49	-1.87	0.92	
OPH - AP	-0.31	0.42	-0.75	1.00	
OPP - AP	-0.37	0.33	-1.10	1.00	
OPU - AP	-0.76	0.33	-2.33	0.67	

OSI - AP	-0.17	0.44	-0.40	1.00	
PB - AP	-0.56	0.35	-1.61	0.98	
PL - AP	-0.92	0.34	-2.73	0.36	
DV - DS	0.13	0.58	0.22	1.00	
GCd - DS	0.18	0.36	0.49	1.00	
GD - DS	-1.07	0.63	-1.70	0.97	
GCt - DS	-0.07	0.48	-0.15	1.00	
NO - DS	0.40	0.39	1.03	1.00	
NU - DS	0.79	0.38	2.08	0.83	
OA - DS	-0.29	0.48	-0.62	1.00	
OMA - DS	-0.70	0.41	-1.71	0.97	
OMI - DS	-0.31	0.35	-0.89	1.00	
OPA - DS	-0.57	0.54	-1.05	1.00	
OPH - DS	0.04	0.48	0.08	1.00	
OPP - DS	-0.01	0.40	-0.03	1.00	
OPU - DS	-0.40	0.40	-1.02	1.00	
OSI - DS	0.18	0.49	0.36	1.00	
PB - DS	-0.20	0.41	-0.50	1.00	
PL - DS	-0.57	0.41	-1.40	1.00	
GCd - DV	0.05	0.54	0.09	1.00	
GD - DV	-1.20	0.75	-1.61	0.98	
GCt - DV	-0.20	0.62	-0.32	1.00	
NO - DV	0.27	0.56	0.49	1.00	
NU - DV	0.67	0.55	1.21	1.00	
OA - DV	-0.42	0.62	-0.68	1.00	
OMA - DV	-0.83	0.57	-1.45	0.99	
OMI - DV	-0.44	0.53	-0.83	1.00	
OPA - DV	-0.70	0.67	-1.04	1.00	
OPH - DV	-0.09	0.62	-0.14	1.00	
OPP - DV	-0.14	0.57	-0.25	1.00	
OPU - DV	-0.53	0.56	-0.95	1.00	
OSI - DV	0.05	0.63	0.08	1.00	
PB - DV	-0.33	0.57	-0.58	1.00	
PL - DV	-0.70	0.57	-1.23	1.00	
GD - GCd	-1.25	0.59	-2.10	0.82	
GCt - GCd	-0.25	0.43	-0.59	1.00	
NO - GCd	0.22	0.33	0.68	1.00	
NU - GCd	0.61	0.32	1.94	0.90	
OA - GCd	-0.47	0.43	-1.11	1.00	
OMA - GCd	-0.88	0.35	-2.51	0.52	
OMI - GCd	-0.49	0.29	-1.72	0.96	
OPA - GCd	-0.75	0.50	-1.50	0.99	
OPH - GCd	-0.14	0.43	-0.33	1.00	

OPP - GCd	-0.19	0.34	-0.56	1.00	
OPU - GCd	-0.58	0.34	-1.74	0.96	
OSI - GCd	2.11e-15	0.45	0.00	1.00	
PB - GCd	-0.38	0.36	-1.08	1.00	
PL - GCd	-0.75	0.35	-2.15	0.79	
GCt - GD	1.00	0.67	1.50	0.99	
NO - GD	1.48	0.61	2.41	0.60	
NU - GD	1.86	0.60	3.08	0.17	
OA - GD	0.78	0.67	1.16	1.00	
OMA - GD	0.38	0.62	0.60	1.00	
OMI - GD	0.76	0.59	1.29	1.00	
OPA - GD	0.50	0.72	0.70	1.00	
OPH - GD	1.11	0.67	1.66	0.97	
OPP - GD	1.06	0.62	1.71	0.97	
OPU - GD	0.67	0.61	1.09	1.00	
OSI - GD	1.25	0.68	1.84	0.94	
PB - GD	0.87	0.63	1.39	1.00	
PL - GD	0.50	0.62	0.80	1.00	
NO - GCt	0.47	0.45	1.05	1.00	
NU - GCt	0.86	0.44	1.96	0.89	
OA - GCt	-0.22	0.52	-0.42	1.00	
OMA - GCt	-0.63	0.46	-1.35	1.00	
OMI - GCt	-0.24	0.42	-0.58	1.00	
OPA - GCt	-0.50	0.59	-0.85	1.00	
OPH - GCt	0.11	0.52	0.21	1.00	
OPP - GCt	0.06	0.46	0.13	1.00	
OPU - GCt	-0.33	0.45	-0.73	1.00	
OSI - GCt	0.25	0.54	0.46	1.00	
PB - GCt	-0.13	0.47	-0.28	1.00	
PL - GCt	-0.50	0.46	-1.08	1.00	
NU - NO	0.39	0.35	1.12	1.00	
OA - NO	-0.70	0.45	-1.55	0.99	
OMA - NO	-1.10	0.38	-2.91	0.25	
OMI - NO	-0.72	0.32	-2.24	0.73	
OPA - NO	-0.97	0.52	-1.87	0.92	
OPH - NO	-0.36	0.45	-0.81	1.00	
OPP - NO	-0.41	0.37	-1.12	1.00	
OPU - NO	-0.81	0.37	-2.21	0.75	
OSI - NO	-0.22	0.47	-0.48	1.00	
PB - NO	-0.61	0.38	-1.58	0.98	
PL - NO	-0.97	0.38	-2.58	0.47	
OA - NU	-1.09	0.44	-2.47	0.56	
OMA - NU	-1.49	0.37	-4.08	**<0.01**	**

OMI - NU	-1.11	0.31	-3.61	**0.04**	*
OPA - NU	-1.36	0.51	-2.66	0.41	
OPH - NU	-0.75	0.44	-1.71	0.97	
OPP - NU	-0.80	0.36	-2.24	0.73	
OPU - NU	-1.20	0.35	-3.39	0.07	.
OSI - NU	-0.61	0.46	-1.34	1.00	
PB - NU	-1.00	0.37	-2.68	0.40	
PL - NU	-1.36	0.37	-3.73	**0.02**	*
OMA - OA	-0.40	0.46	-0.87	1.00	
OMI - OA	-0.02	0.42	-0.05	1.00	
OPA - OA	-0.28	0.59	-0.47	1.00	
OPH - OA	0.33	0.52	0.64	1.00	
OPP - OA	0.28	0.46	0.61	1.00	
OPU - OA	-0.11	0.45	-0.25	1.00	
OSI - OA	0.47	0.54	0.87	1.00	
PB - OA	0.09	0.47	0.19	1.00	
PL - OA	-0.28	0.46	-0.60	1.00	
OMI - OMA	0.38	0.34	1.13	1.00	
OPA - OMA	0.13	0.53	0.24	1.00	
OPH - OMA	0.74	0.46	1.59	0.98	
OPP - OMA	0.68	0.39	1.77	0.95	
OPU - OMA	0.29	0.38	0.76	1.00	
OSI - OMA	0.88	0.48	1.82	0.94	
PB - OMA	0.49	0.40	1.23	1.00	
PL - OMA	0.13	0.39	0.32	1.00	
OPA - OMI	-0.26	0.49	-0.52	1.00	
OPH - OMI	0.35	0.42	0.85	1.00	
OPP - OMI	0.30	0.33	0.91	1.00	
OPU - OMI	-0.09	0.33	-0.28	1.00	
OSI - OMI	0.49	0.44	1.12	1.00	
PB - OMI	0.11	0.35	0.32	1.00	
PL - OMI	-0.26	0.34	-0.76	1.00	
OPH - OPA	0.61	0.59	1.04	1.00	
OPP - OPA	0.56	0.53	1.06	1.00	
OPU - OPA	0.17	0.52	0.32	1.00	
OSI - OPA	0.75	0.60	1.25	1.00	
PB - OPA	0.37	0.54	0.68	1.00	
PL - OPA	0.00	0.53	0.00	1.00	
OPP - OPH	-0.05	0.46	-0.11	1.00	
OPU - OPH	-0.44	0.45	-0.98	1.00	
OSI - OPH	0.14	0.54	0.26	1.00	
PB - OPH	-0.24	0.47	-0.52	1.00	
PL - OPH	-0.61	0.46	-1.32	1.00	

OPU - OPP	-0.39	0.38	-1.04	1.00	
OSI - OPP	0.19	0.48	0.40	1.00	
PB - OPP	-0.19	0.39	-0.49	1.00	
PL - OPP	-0.56	0.39	-1.44	0.99	
OSI - OPU	0.58	0.47	1.24	1.00	
PB - OPU	0.20	0.39	0.52	1.00	
PL - OPU	-0.17	0.38	-0.44	1.00	
PB - OSI	-0.38	0.49	-0.79	1.00	
PL - OSI	-0.75	0.48	-1.56	0.99	
PL - PB	-0.37	0.40	-0.92	1.00	

., *, ** and *** indicate P values of > 0.05, < 0.05, ≤ 0.01 and ≤ 0.001, respectively.

Table S5. Distance-based Redundance analysis results on the effect of locality and orchid host identity on the fungal communities in plants of 11 orchid species that occurred at least in two sites. Results of (a) distance-RDA testing the effects of locality (i.e., site) and orchid host identity together, (b) marginal db-RDA testing the effect of each variable individually, and (c) conditional partial db-RDA with the covariates.

Factors	(a) Db-RDA			
	R^2 adj (%)	F value	Df	P value
Orchid host	33.42	10.08	10	**0.001**
Locality		11.07	3	**0.001**

Factors	(b) Marginal db-RDA			
	R^2 adj (%)	F value	Df	P value
Orchid host	27.65	10.21	10	**0.001**
Locality	8.03	8.01	3	**0.001**

Factors	(c) Partial db-RDA				Covariates
	R^2 adj (%)	F value	Df	P value	
Orchid host	25.39	10.08	10	**0.001**	Locality
Locality	5.77	7.67	3	**0.001**	Orchid host

Table S6. ANOVA multiple comparisons of the mean Shannon, intersite and interspecies dissimilarity indices of rhizoctonian fungal communities. **A.** Anova test comparing the mean Shannon diversity, intersite (intersite DI) and interspecies (interspecies DI) dissimilarity indices for orchid species occurring at least in two sites, and **B.** Tukey's Post-hoc tests comparing the mean Shannon diversity and intersite DI between orchid species.

A. Anova test

Shannon index	Df	SumSq	MeanSq	F value	Pr(>F)	Sign.
Species	10	0.87	0.09	3.44	**0.01**	*
Residuals	17	0.43	0.03			

Intersite DI	Df	SumSq	MeanSq	F value	Pr(>F)	Sign.
Species	10	1.71	0.17	38.85	**1.0e-09**	***
Residuals	17	0.07	0.004			

Interspecies DI	Df	SumSq	MeanSq	F value	Pr(>F)	Sign.
Species	10	0.04	0.004	0.71	0.71	
Residuals	17	0.10	0.006			

B. Tukey multiple comparisons between orchid species

**Shannon diversity**						**Intersite dissimilarity**					
Species	Estimate	Std.Error	t	P value	Sign.	Species	Estimate	Std.Error	t	P value	Sign.
AP - AM	-0.13	0.13	-1.00	0.99		AP - AM	-0.29	0.05	-5.27	**<0.01**	**
DS - AM	-0.33	0.14	-2.30	0.47		DS - AM	0.11	0.06	1.77	0.78	
GCd - AM	-0.24	0.13	-1.86	0.73		GCd - AM	0.14	0.05	2.60	0.31	
NO - AM	-0.13	0.13	-1.02	0.99		NO - AM	0.02	0.05	0.45	1.00	
NU - AM	0.08	0.13	0.59	1.00		NU - AM	0.09	0.05	1.62	0.85	
OMA - AM	-0.54	0.14	-3.74	**0.04**	*	OMA - AM	-0.66	0.06	-10.95	**<0.01**	***
OMI - AM	-0.36	0.13	-2.78	0.24		OMI - AM	-0.06	0.05	-1.18	0.98	
OPP - AM	-0.27	0.15	-1.88	0.72		OPP - AM	-0.06	0.06	-1.06	0.99	
OPU - AM	-0.44	0.15	-3.04	0.16		OPU - AM	-0.57	0.06	-9.39	**<0.01**	***
PB - AM	-0.30	0.15	-2.08	0.60		PB - AM	0.11	0.06	1.80	0.77	
DS - AP	-0.20	0.15	-1.40	0.93		DS - AP	0.39	0.06	6.49	**<0.01**	***
GCd - AP	-0.11	0.13	-0.86	1.00		GCd - AP	0.43	0.05	7.88	**<0.01**	***
NO - AP	-0.002	0.13	-0.02	1.00		NO - AP	0.31	0.05	5.72	**<0.01**	***
NU - AP	0.21	0.13	1.59	0.86		NU - AP	0.37	0.05	6.89	**<0.01**	***
OMA - AP	-0.41	0.15	-2.85	0.22		OMA - AP	-0.38	0.06	-6.24	**<0.01**	***
OMI - AP	-0.23	0.13	-1.77	0.78		OMI - AP	0.22	0.05	4.09	**0.02**	*

OPP - AP	-0.14	0.15	-0.98	0.99		OPP - AP	0.22	0.06	3.65	0.05	.
OPU - AP	-0.31	0.15	-2.15	0.56		OPU - AP	-0.28	0.06	-4.68	**<0.01**	**
PB - AP	-0.17	0.15	-1.18	0.98		PB - AP	0.39	0.06	6.51	**<0.01**	***
GCd - DS	0.09	0.15	0.63	1.00		GCd - DS	0.03	0.06	0.56	1.00	
NO - DS	0.20	0.15	1.39	0.94		NO - DS	-0.08	0.06	-1.37	0.94	
NU - DS	0.41	0.15	2.83	0.23		NU - DS	-0.02	0.06	-0.32	1.00	
OMA - DS	-0.21	0.16	-1.32	0.95		OMA - DS	-0.77	0.07	-11.62	**<0.01**	***
OMI - DS	-0.03	0.15	-0.18	1.00		OMI - DS	-0.17	0.06	-2.83	0.23	
OPP - DS	0.06	0.16	0.38	1.00		OPP - DS	-0.17	0.07	-2.59	0.32	
OPU - DS	-0.11	0.16	-0.68	1.00		OPU - DS	-0.68	0.07	-10.19	**<0.01**	***
PB - DS	0.03	0.16	0.20	1.00		PB - DS	0.001	0.07	0.02	1.00	
NO - GCd	0.11	0.13	0.84	1.00		NO - GCd	-0.12	0.05	-2.15	0.56	
NU - GCd	0.32	0.13	2.45	0.39		NU - GCd	-0.05	0.05	-0.98	0.99	
OMA - GCd	-0.30	0.15	-2.08	0.60		OMA-GCd	-0.80	0.06	-13.29	**<0.01**	***
OMI - GCd	-0.12	0.13	-0.91	1.00		OMI - GCd	-0.20	0.05	-3.79	**0.04**	*
OPP - GCd	-0.03	0.15	-0.21	1.00		OPP - GCd	-0.21	0.06	-3.39	0.09	.
OPU - GCd	-0.20	0.15	-1.38	0.94		OPU - GCd	-0.71	0.06	-11.73	**<0.01**	***
PB - GCd	-0.06	0.15	-0.41	1.00		PB - GCd	-0.03	0.06	-0.53	1.00	
NU - NO	0.21	0.13	1.61	0.86		NU - NO	0.06	0.05	1.17	0.98	
OMA - NO	-0.41	0.15	-2.83	0.22		OMA - NO	-0.69	0.06	-11.36	**<0.01**	***
OMI - NO	-0.23	0.13	-1.76	0.79		OMI - NO	-0.09	0.05	-1.63	0.85	
OPP - NO	-0.14	0.15	-0.97	0.99		OPP - NO	-0.09	0.06	-1.46	0.91	
OPU - NO	-0.31	0.15	-2.13	0.57		OPU - NO	-0.59	0.06	-9.80	**<0.01**	***
PB - NO	-0.17	0.15	-1.17	1.00		PB - NO	0.08	0.06	1.39	0.93	
OMA - NU	-0.62	0.15	-4.27	**0.02**	*	OMA - NU	-0.75	0.06	-12.41	**<0.01**	***
OMI - NU	-0.44	0.13	-3.37	0.09	.	OMI - NU	-0.15	0.05	-2.80	0.23	
OPP - NU	-0.35	0.15	-2.41	0.41		OPP - NU	-0.15	0.06	-2.51	0.36	
OPU - NU	-0.52	0.15	-3.57	0.06	.	OPU - NU	-0.66	0.06	-10.85	**<0.01**	***
PB - NU	-0.38	0.15	-2.61	0.31		PB - NU	0.02	0.06	0.35	1.00	
OMI - OMA	0.18	0.15	1.26	0.96		OMI - OMA	0.60	0.06	9.90	**<0.01**	***
OPP - OMA	0.27	0.16	1.70	0.81		OPP - OMA	0.60	0.07	9.03	**<0.01**	***
OPU - OMA	0.10	0.16	0.64	1.00		OPU - OMA	0.09	0.07	1.42	0.92	
PB - OMA	0.24	0.16	1.52	0.89		PB - OMA	0.77	0.07	11.64	**<0.01**	***
OPP - OMI	0.09	0.15	0.60	1.00		OPP - OMI	-0.0003	0.06	-0.004	1.00	
OPU - OMI	-0.08	0.15	-0.56	1.00		OPU - OMI	-0.50	0.06	-8.34	**<0.01**	***
PB - OMI	0.06	0.15	0.40	1.00		PB - OMI	0.17	0.06	2.85	0.22	
OPU - OPP	-0.17	0.16	-1.06	0.99		OPU - OPP	-0.50	0.07	-7.61	**<0.01**	***
PB - OPP	-0.03	0.16	-0.18	1.00		PB - OPP	0.17	0.07	2.61	0.31	
PB - OPU	0.14	0.16	0.88	1.00		PB - OPU	0.68	0.07	10.22	**<0.01**	***

*, ** and *** indicate P values of < 0.05, ≤ 0.01 and ≤ 0.001, respectively.

Table S7. Details on the number of fungal OTUs, plants, roots, and culture plates in each studied orchid species. The numbers of OTUs are given regarding the detection methods: culture-independent only (C-IND), culture-dependent only (C-D) or both methods. The total number of OTUs represents the fungal richness. Additional information refers to the numbers of i) plants used for NGS and from which fungal strains were cultured, ii) roots containing cultured strains, and iii) Petri dishes with cultivated strains identified as rhizoctonia, non-targeted fungi or contamination (named “other cultures”). The last row represents total numbers.

Orchid species	Site	OTUs (C-IND)	OTUs (C-D)	OTUs (both methods)	Fungal richness	Plants in NGS	Plants with culture	Roots with culture	Rhizoctonia cultures	Other cultures
**AM**	FR1	9	0	2	11	8	8	17	37	0
	FR2	6	1	5	12	7	9	17	48	7
	CZ1	9	0	3	12	12	6	11	70	1
**AP**	FR1	9	0	4	13	14	7	20	44	8
	FR2	0	0	3	3	10	6	11	17	2
	CZ2	7	1	4	12	9	6	12	18	3
**DS**	FR1	2	0	2	4	5	4	8	28	0
	CZ1	6	0	1	7	9	2	5	18	8
**DV**	FR1	5	0	2	7	5	6	11	24	2
**GCd**	FR1	11	0	0	11	11	2	3	0	2
	CZ1	8	0	2	10	9	8	29	123	0
	CZ2	8	1	1	10	8	6	10	2	5
**GCt**	CZ2	6	0	2	8	9	7	15	4	10
**GD**	CZ2	1	0	0	1	4	2	3	0	2
**NO**	FR1	12	0	1	13	7	4	6	1	4
	CZ1	3	0	4	7	6	4	7	9	2
	CZ2	8	0	1	9	7	3	4	6	6
**NU**	FR1	7	0	3	10	5	3	4	10	1
	FR2	9	2	3	14	10	4	6	15	0
	CZ2	7	0	3	10	7	7	21	57	1
**OPA**	CZ2	3	0	1	4	6	5	13	16	3
**OPH**	CZ2	4	0	1	5	9	5	7	3	6
**OPP**	FR1	6	1	1	8	10	5	8	11	3
	FR2	3	0	3	6	7	4	5	10	1
**OA**	FR2	6	0	1	7	9	2	3	7	2
**OMA**	FR1	2	0	1	3	7	3	5	9	1
	FR2	2	0	1	3	9	6	11	9	2
**OMI**	FR1	7	0	0	7	10	0	0	0	0
	FR2	3	0	0	3	10	1	3	0	3
	CZ2	9	0	0	9	13	4	6	0	5
**OPU**	FR1	5	0	1	6	10	1	1	4	0
	FR2	3	0	0	3	8	0	0	0	0
**OSI**	FR2	6	0	1	7	8	1	3	14	0
**PB**	CZ1	2	0	5	7	10	10	28	95	0
	CZ2	2	0	3	5	5	5	19	63	1
**PL**	FR1	4	0	2	6	6	6	15	29	0
	FR2	2	0	3	5	10	10	23	95	0
**Total**	-	54	0	30	54	309	172	370	896	91

Supplementary figures:

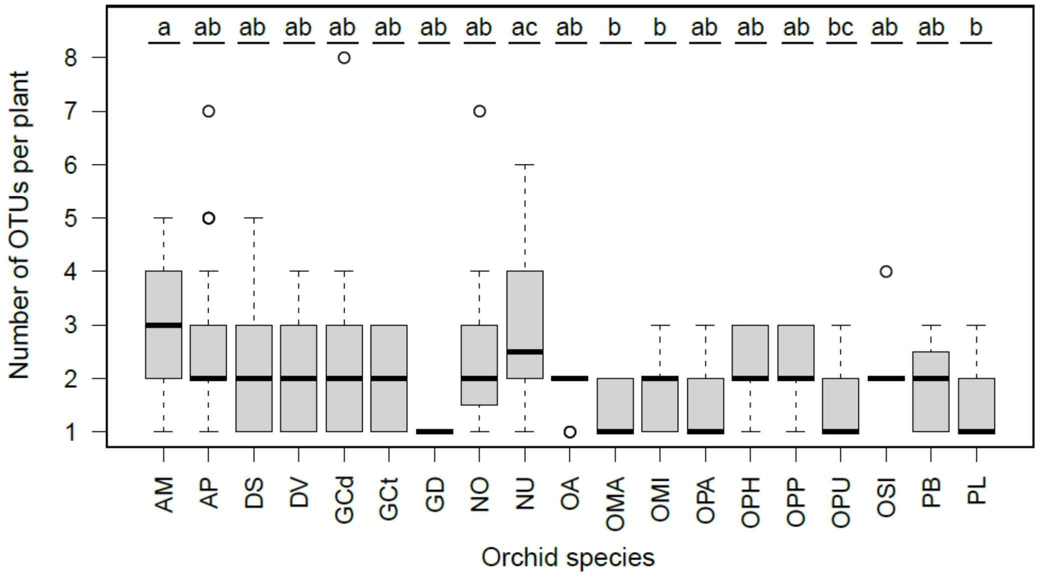

Figure S1. Rhizoctonian OTUs at individual plant level in the 19 studied orchid species. The boxes represent median, upper, and lower quartiles with the whiskers showing minimal and maximal values, and outliers in circle, in the number of OTUs found among orchid plants. Different letters denote significant differences in the number of OTUs between orchid species at $P < 0.05$ based on the Tukey tests following ANOVA. Orchid species: *Anacamptis morio* (AM), *A. pyramidalis* (AP); *Dactylorhiza sambucina* (DS), *D. viridis* (DV); *Gymnadenia conopsea* diploid (GCd), *G. conopsea* tetraploid (GCt), *G. densiflora* (GD)*; Neottia ovata* (NO)*; Neotinea ustulata* (NU); *Ophrys apifera* (OPA), *Op. holubyana* (OPH), *Op. sphegodes subsp. passionis* (OPP); *Orchis anthropophora* (OA), *O. mascula* (OMA), *O. militaris* (OMI), *O. purpurea* (OPU), *O. simia* (OSI); and *Platanthera bifolia* (PB), *Platanthera spp.* (PL). See Table S4-B for details.
